# A qualitative study of geriatric specialist nurses’ experiences to navigate delirium in the elderly

**DOI:** 10.1186/s12912-024-02100-x

**Published:** 2024-06-25

**Authors:** Mei Wu, Zhen Chen, Yamin Xu, Liting Zhao, Lirong Zhao, Lu Xia

**Affiliations:** 1 Department of nursing, Huadong Hospital Affiliated to Fudan University, Shanghai, China; 2 Day Surgery Unit, Huadong Hospital Affiliated to Fudan University, Shanghai, China; 3 Surgical Intensive Care Unit, Huadong Hospital Affiliated to Fudan University, Shanghai, China; 4 Day Care Chemotherapy, Huadong Hospital Affiliated to Fudan University, Shanghai, China; 5 Department of Neurology, Huadong Hospital Affiliated to Fudan University, Shanghai, China

**Keywords:** Delirium, Elderly, Nurse, Qualitative, Care, Nursing

## Abstract

**Background:**

The experiences and perceptions of geriatric specialist nurses are pivotal to understanding the complexities of managing delirium and to developing effective nursing interventions. This qualitative study aims to explore these experiences and perceptions to inform the enhancement of clinical geriatric nursing and care practices.

**Methods:**

Utilizing a qualitative exploratory design, this research engaged a convenience sample of geriatric specialist nurses at a tertiary hospital in Shanghai, China through focus groups and semi-structured interviews. Data were rigorously analyzed using Colaizzi’s phenomenological method, which facilitated the identification of themes that emerged from the narratives of the geriatric specialist nurses.

**Results:**

The thematic analysis yielded three major themes that encapsulate the nurses’ experiences and perceptions. Theme 1: Understanding of Delirium, highlighted the nurses’ awareness of the condition’s significance, yet it was often deprioritized due to the pressing demands of managing more acute and immediately life-threatening conditions. Theme 2: Barriers in Application, brought to light the multifaceted challenges faced by nurses, including language barriers, the frequency and consistency of delirium assessments, the social determinants of health, and the nurses’ own competencies in assessment. Theme 3: Evolution of Nursing Approaches, detailed the adaptive strategies employed by nurses, such as managing nursing adverse events, improving communication with patients’ families, and adopting a proactive stance towards long-term patient outcomes.

**Conclusions:**

The findings suggest that while geriatric specialist nurses recognize the importance of delirium assessment, there are several barriers to effective application. The study underscores the imperative for the advancement of more refined delirium assessment and care protocols, tailored to address the unique requirements of geriatric nursing care.

**Supplementary Information:**

The online version contains supplementary material available at 10.1186/s12912-024-02100-x.

## Introduction

Delirium is characterized as a clinical syndrome encompassing acute disturbances in consciousness, attention, and cognitive or perceptual functions, which are subject to fluctuations throughout the day. This transient neuropsychiatric condition is marked by a rapid onset and variability in symptom presentation, reflecting the dynamic nature of the underlying pathophysiological processes [1, 2]. Postoperative delirium emerges as a prevalent postoperative complication, predominantly affecting the geriatric population. The reported incidence among elderly patients varies widely, from 5 to 50%, with specific procedures such as spinal and joint surgeries exhibiting a notably higher rate of approximately 15.2% [3, 4]. In the demographic of patients over 65 years of age who have undergone non-cardiac surgeries, the incidence of postoperative delirium is observed to range between 6.1% and 57.1%, culminating in an aggregate rate of 11.1% [5]. Among the elderly who have sustained a hip fracture, the onset of delirium within the initial 1 to 3 postoperative days is particularly concerning, with reported incidence rates as high as 17–61% [6, 7], underscoring the heightened vulnerability of those who have undergone intricate and urgent surgical interventions. Delirium within the Intensive Care Unit (ICU) settings present a significant clinical challenge, with an estimated 35% of ICU patients experiencing misdiagnosis or diagnostic oversight concerning delirium [8, 9]. The ICU environment, characterized by its distinctive conditions, may be particularly conducive to the development of delirium. The incidence of delirium in ICU patients is influenced by a multitude of factors, including but not limited to age, type of surgery, the elective or emergency nature of the procedure, and the utilization of various delirium assessment instruments [10, 11]. The collective data underscore the imperative need for vigilant monitoring and proactive management of postoperative delirium, particularly in the context of elderly patients and ICU environments [12, 13]. The implementation of preventive strategies and the prompt recognition of delirium are paramount to optimizing patient prognoses and averting adverse outcomes [14].

However, its low recognition rate is not consistent with the high incidence rate. The low recognition rate of delirium in hospitals [15] around the world has become an important issue, and medical staff’ recognition rate of delirium is less than 25% [16–18]. The unrecognized rate of delirium (defined as delirium not determined by the patient’s therapist and nurse) is about 33-95% [19]. The low recognition rate is due to its lack of symptomatic characteristics [20]and the variability of symptoms [18]. The identification of delirium is a huge challenge for nurses. Trogrlic et al. [21]summarized the literature on delirium from 2000 to 2010, and found that the cognitive and attitude level of delirium in ICU such as medical staff the main obstacle affecting delirium identification. Inouye et al. [22] found that the accuracy rate of nurses in identifying delirium in 797 patients was only 31% of that of researchers. Augmenting the proficiency of healthcare providers in the recognition of delirium is of paramount importance. The accurate and timely identification of delirium is a critical component in the continuum of care for patients at risk. Enhancing providers’ ability to detect delirium can lead to earlier intervention, which is instrumental in mitigating the severity and duration of the episode, thereby potentially improving patient outcomes and reducing healthcare-associated complications.

Delirium assessment in hospitals can help healthcare professionals to manage patient safety, which is of great significance for some critically ill patients and elderly patients [23]. Previous studies have reported that Individuals with delirium are found to have a higher mortality rate, extended hospital stays, and increased medical expenditures compared to their elderly counterparts who are free from delirium [24–26]. Delirium, when it occurs, is often overlooked by nursing staff, particularly when they are not well-versed in the nuances of delirium assessment and have limited experience with the application of standardized delirium assessment tools. This lack of expertise can lead to a diminished capacity to recognize and respond to the signs of delirium, thereby affecting the quality of care and the clinical outcomes for affected patients. The underdiagnosis of delirium not only reflects a gap in clinical perception but also highlights the need for targeted education and training to enhance nurses’ competencies in this critical area of geriatric care [27, 28]. The study surveyed the real-world uptake of delirium detection methods has shown that the Confusion Assessment Method was used in 65/146 (45%) units and 4AT was with 80% of units reporting use in the UK [29]. The significance of comprehensive delirium assessment in effective delirium management is increasingly acknowledged within the medical community. However, the empirical understanding of nurses’ experiences, particularly in relation to the challenges and ambiguities encountered during delirium assessment, remains under-explored.

## Theoretical framework

Colaizzi’s phenomenological approach is a widely utilized data analysis technique in qualitative research, particularly within the realm of phenomenological studies [30]. The goal of this method is to uncover the intrinsic meanings and essence of individuals’ lived experiences through a deep understanding [31]. The significance of the Colaizzi method lies in its structured approach to analyzing qualitative data, assisting researchers in revealing the profound experiences and sentiments of participants [32]. Moreover, the extension and adaptation of this method can enhance the rigor of research and broaden the scope of information sources, allowing for a more in-depth depiction of the phenomena under study. This not only aids in elevating the quality and depth of research but also offers richer and more concrete guidance for nursing practice. A qualitative inquiry into the authentic experiences of nurses as they engage in the assessment process is of considerable value. Such exploration can yield profound insights, ultimately contributing to the refinement of delirium assessment methodologies and the enhancement of clinical practice in geriatric care [33].

### Objectives

Therefore, the present study was designed to systematically evaluate the experiences and perceptions of geriatric nurse specialists regarding the management of delirium in elderly patients within the specialized domains of postoperative care and intensive care units. The objective was to generate empirical evidence that can significantly inform and enhance the standards of geriatric nursing care in these critical healthcare settings.

## Methods

### Design

A prospective qualitative research methodology was employed, utilizing focus group interviews in conjunction with the Colaizzi’s phenomenological approach [34], to elicit an in-depth understanding of the experiences and perceptions of geriatric specialist nurses regarding the phenomenon of delirium in elderly patients within the context of postoperative care and intensive care units. This methodological choice was selected to capture a comprehensive and nuanced description of the nurses’ encounters with delirium, providing a rich dataset that can inform the development of targeted interventions and enhance the quality of geriatric care in these critical settings.

### Settings and participants

The study sample was recruited from geriatric specialist nurses in the postoperative care and intensive care units of a tertiary hospital in Shanghai, China. Objective sampling was used to select geriatric specialist nurses who had the greatest possible experience and perceptions. The criteria for inclusion in this study were geriatric specialist nurses who were able to independently complete a delirium assessment after 2 months of delirium assessment. Nurses were identified and recruited through postoperative care and intensive care units assessed by researchers for delirium. If they agreed to participate, their contact information would be sent to the research team. The number of participants in the focus group reached saturation when there were no more new theme to be found during the conversations. The researcher was a different person to undertake the focus groups interview. After 3 focus group interviews, 19 nurses completed the focus group interviews.

### Data collection

During the interview, the participants read the outline of the study and were informed by the researcher of the purpose and process of the study, the risks, and so on. They pointed out that participation was voluntary and received oral consent and recording permission.

The primary investigator (WM), executed all interviews with meticulous adherence to the study protocol. A total of three focus groups were convened, comprising 19 geriatric nurse specialists who contributed their expertise to the qualitative inquiry. These focus group interviews were strategically scheduled across three distinct time points to capture the temporal dynamics of the participants’ experiences and insights within the study’s framework. Conducting multiple focus group discussions enriches the dataset, facilitating a profound comprehension of the research subject. As time progresses, the rapport and trust among participants and with the researchers are likely to intensify, fostering an environment conducive to more candid and forthright dialogues. Subsequent discussions serve to deepen the investigator’s grasp of the perspectives and experiences shared by the participants, particularly in areas that may have emerged as requiring further elucidation or exploration post the initial session.

Based on the existing research and clinical experience of the study group, an interview guide (supplementary file 1) was developed for geriatric specialist nurses to assess their delirium experiences and perceptions. Interview questions were as follows: (I)How did you observe delirium? What was the priority of delirium in clinical nursing? (II)Did the current assessment form help you identify patients with delirium more easily? What were the problems and puzzles in the process of assessment? (III)Whether other ways did you think can help you? Show us some examples. (IV)Identify whether delirium affects patients’ outcomes. The problem was developed by the first author and examined by the project team. A small number of geriatric specialist nurses were tested in advance. Interviews were conducted with geriatric specialist nurses who were assessed for delirium for 2 months. The average length of each group interview was about 45 min. Voice record data was transcribed and saved as a text document. Every interview would record the notes on the scene to record the introspection and preliminary ideas. All the researchers received qualitative research training in qualitative research data collection methods, interview skills and analysis methods in advance, and carried out pre-experimental analysis to avoid the deviation of research results caused by qualitative research skills. All data must be analyzed by two researchers at the same time to avoid the deviation of the researchers’ own values and emotional integration in the process of data collection and analysis.

### Data analysis

Upon the conclusion of each interview session, a meticulous process of data consolidation was initiated. Two researchers dedicated a full 24-hour period to meticulously reviewing the audio recordings, ensuring that every nuance and detail was captured with accuracy. This iterative listening process was conducted to enhance the depth of understanding and to identify the subtleties that might be present in the spoken narratives. Simultaneously, the researchers cross-referenced their comprehensive written notes taken during the interviews. These notes served as a valuable resource for contextualizing the verbal exchanges and for augmenting the transcription process. The transcription of the recordings was approached with the utmost rigor, striving to convert the spoken word into a written format that remained faithful to the original dialogues. In the present study, the data derived from the focus group interviews were meticulously analyzed using Colaizzi’s phenomenological approach, a well-established method for qualitative data analysis. This process involved several systematic steps to ensure a rigorous and authentic interpretation of the participants’ experiences. Initially, the interviews were transcribed verbatim, capturing every detail of the dialogue. The transcriptions were then thoroughly read and re-read to achieve a deep immersion in the data. Colaizzi’s data analysis focused on integrating the feelings of the interviewees, adding the steps for the researchers to analyze the conclusions, which was divided into seven stages: (1) detailed recording and careful repeated reading of all materials; (2) extraction of meaningful statements; (3) induction and extraction of meaning from repeated and important statements; (4) searched for common concepts or characteristics of meaning to form themes, topic groups and categories; (5) related the topic to the research phenomenon to make a complete description; (6) identified similar viewpoints; (7) returned the results to the interviewees to verify the authenticity of the content.

### Ethical considerations

The study received approval from the Ethics Committee of Huadong Hospital, with the assigned approval number No.2018K051. Prior to the commencement of the study, the researcher engaged in dialogue with the participants, elucidating the study’s objectives, methodology, importance, and the assurance of confidentiality. A foundation of trust was established, and participants were informed of their voluntary nature of participation. They were assured that they could withdraw from the interview at any juncture. Additionally, participants were requested to provide their informed consent by signing a consent form. Each participant was represented by a numeric code.

## Results

Nineteen geriatric specialist nurses (eighteen females and one male), with a median of 31 years old were recruited. The range of cumulative working experience was 2–26 years, and the range of experience of working on the surgical ward or ICU was 1–22 years. Characteristics of the participants are showed in Table 1.

**Table 1:** The characteristics of included nurses(*n* = 19)

Variable	*n*	%	Mean	SD
Gender				
Female	18	94.7		
Male	1	5.3		
Age(years)			31.4	5.1
25–35	16	84.2		
36–40	1	5.3		
≥41	2	10.5		
Length of tenure (years)			9.9	6.3
1–5	3	15.8		
6–10	10	52.6		
≥11	6	31.6		
Education				
Diploma	3	15.8		
Bachelor’s	16	84.2		
Length of surgical service (years)			7.8	5.6
1–5	7	36.8		
6–10	6	31.6		
≥11	6	31.6		
Hospital care Unit				
General surgery	7	36.8		
ICU	7	36.8		
Orthopedics	5	26.4		

The thematic analysis conducted in this study has systematically identified three principal themes that encapsulate the comprehensive experiences and perceptions of the nurses.

###  Understanding of delirium

Delirium is an important part of nursing cognition. However, the priority of acute and life-threatening is still the focus of health care. *Nurse G1N2: “I know delirium can result from bad outcomes, but in the immediate care of patients, these threats, such as extubation, elevated body temperature, and altered vital signs, are still issues we need to address immediately.”*

Before clinical training, nurses mainly rely on their own clinical experience to judge the occurrence of delirium. The content of the assessment includes the state of consciousness, lack of complete assessment, and did not understand the assessment scale of delirium. *Nurse G1N1: “If a patient is told not to remove the oxygen saturation clip after surgery, and if we have talked to him three times and he still wanted to remove it, we would consider his delirious problem”; Nurse G1N2: " We will inquire of the patients who are not on ventilators with questions like “Where are you?” and “What surgery have you undergone?” If a patient is unable to respond, we will suspect the presence of delirium. “*

Subjective assessments carry the inherent risk of introducing bias and error into the assessment process, a phenomenon that is particularly pertinent in the context of delirium, where the condition’s subtle manifestations can elude immediate clinical detection. The nuanced nature of delirium, especially in its early or mild stages, often presents a formidable challenge to healthcare providers, who may struggle to discern its presence amidst the myriad clinical presentations encountered in patient care. *Nurse G1N4: “If a patient is quiet and doesn’t evaluate, it’s hard for us to detect delirium relative to those who are very upset.”*

###  Barriers in application

####  Language

In the process of using the scale, the assessment methods and tone of nurses may lead to differences in patients’ understanding. Moreover, as far as the problems on the assessment form are concerned, they are not routine enough and divorced from the actual problems, which makes it difficult for some elderly patients to understand their educational level. *Nurse G1N7: “Some patients come from other provinces and municipalities, there are language communication barriers if the assessment is wrong because they do not understand, problems will occur in the accuracy of delirium assessment.” Nurse G2N10: “Some patients are not well educated enough to answer questions, and the results are controversial.” Nurse G1N5: “Can we judge delirium by asking a patient how many children they have so that it’s closer to the patient’s daily life?”*

####  Frequency of assessment

Clinically, nurses evaluate patients with delirium according to the frequency of assessment recommended by the scale, but nurses have questions about it. *Nurse G1N3: “After the surgery, I did the first-time assessment. The patient was able to answer correctly. After three times, the patient questioned my assessment and rejected my assessment. Is it necessary for at least nine full assessments in this situation?” Nurse G3N15: “I think at the first time, delirium did not occur, followed by other communication abnormalities, then it’s time to assess whether more appropriate.”*

The intensity and frequency of postoperative delirium assessments for nurses will be adjusted based on the changes in the patient’s condition. *Nurse G2N9*: “*When I suspect a patient is experiencing delirium, I will immediately report to the physician and pay closer attention to any changes in the patient’s condition”.*

####  Social problems

Family members may lack comprehensive insight into the cognitive status of their elderly relatives prior to institutionalization, or they may misconstrue early signs of cognitive impairment as an inevitable aspect of the aging process. This misperception, coupled with the complexities of geriatric care, amplifies the challenges faced by nursing staff in the postoperative assessment of delirium. The task of accurately diagnosing postoperative cognitive changes is further complicated by the potential underestimation of pre-existing cognitive deficits by family members and the inherent variability in the presentation of delirium symptoms. *Nurse G2N11: “Family members told us that their parents are well when explaining the patient’s cognitive status, but in fact, on admission, we assess that there is a certain cognitive dysfunction, so it is difficult to distinguish dementia from delirium, which makes it more difficult to assess delirium.”*

####  The assessment competence of nurses

Although nurses were trained in professional delirium knowledge and assessment tools (supplementary file 2 and 3), the use of tools still depends on the professional clinical competence of nurses. For some junior nurses, there is a certain ability requirement, and the accuracy of the results may have deviated. *Nurse G2N9: “The patient is in a state of sedation and ignores you in the ward. For some inexperienced nurses, they will think that the patient can cooperate with the treatment nurse well, and there is no delirium problem.”*

###  Changes in the nursing pattern

####  Nursing adverse events

Nurses found in the clinical work that the changes in patients’ condition easily result from delirium, which may lead to patients with unplanned extubation events, especially in some restless patients. These bring the improvement of nursing difficulty. By using the delirium assessment scale to assess the presence of delirium, nursing staff can protect the patient from extubation events. *Nurse G3N13: “There was a patient who was able to answer questions when I evaluated him before, but suddenly in an evening assessment, he couldn’t answer my question and we were protective against him for fear of extubation.”*

####  Communication with family members

Through the delirium assessment of patients, nurses pay more attention to effective communication between patients and family members of patients, educating patients to cooperate with nursing operations, enhancing the sense of security in the ward and confidence in disease rehabilitation. *Nurse G2N8: “Because of illness, patients cannot stay and communicate with their family members for the time being, and family members also difficult to understand the actual situation of patients. After careful explanation of the assessment results, some changes have taken place between patients and family members in the care of patients with treatment.”*

####  Avoiding disputes

For many ICU patients, because of the ward requirements, the patients’ families can not accompany them every time. The changes in the patient’s condition cannot be recognized by their families, and they will have a sense of distrust of the medical staff, especially when there appear to be unrelated cognitive problems with the disease. Family members cannot understand so it is easy to cause medical disputes. Through delirium assessment, nurses are aware of the changes in cognitive status, they can communicate with the family members of patients promptly, explain the problem, and get an understanding of the family members. *Nurse G2N12: “When we communicate with family members of patients without delirium assessment, it is always difficult for us to explain the mental changes of patients, and through assessment, we can explain the changes of patients to family members carefully, so that family members can understand more and avoid disputes.”*

####  Focusing on long-term complications

Delirium assessment enables nurses to realize that delirium in the ward may lead to changes in cognitive function in patients later, and it is very important for the quality of life of patients. Nurse *G1N2: “Our ward is less likely to focus on continuing care after a month, two months, and three months… but if delirium occurs during hospitalization, we find that patients stay in hospital significantly longer, and later recovery is slower than patients with the same disease.”*

## Discussions

In our study, geriatric specialist nurses have a deep understanding of delirium and delirium assessment in their daily work, and a new understanding of previously unnoticed or neglected changes in cognitive status. While the immediate threat of delirium may not be perceived as the foremost concern, nurses have reported observing acute changes in vital signs, such as “elevated body temperature, increased heart rate, and decreased oxygen saturation,” which take precedence in their clinical assessments. This observation aligns with existing literature, indicating that nurses often regard delirium as a lower-priority issue within the ICU setting. The prevailing belief is that delirious patients are not typically viewed as being in significant discomfort, as they may not necessitate specialized treatment or support beyond standard care protocols. Although delirium assessment is incorporated into the overall physical examination, it is not commonly afforded the highest priority by healthcare professionals. This perspective may stem from the assumption that delirium does not warrant the same level of immediate intervention as other critical conditions presenting with more overtly alarming symptoms [35]. At the same time, 50% of nurses give priority to young adults with mixed delirium, and the last priority is inefficient delirium in older patients [36, 37].

There were various barriers to the clinical use of the delirium scale, including language, educational level of the population, frequency of assessment, social problems, and nurses’ assessment ability. It has been reported that assisting the elderly residing in nursing homes is essential to fulfill their emotional and psychological needs, thereby safeguarding and enhancing their spiritual well-being [38, 39]. Although the scale has been localized and localized in Chinese, there are still many practical problems in clinical use. The deficiency in nurses’ knowledge regarding delirium is a pivotal issue that significantly hampers heir assessment capabilities, as evidenced by recent studies [40, 41]. This underscores the necessity for incorporating delirium knowledge training into the routine professional development curriculum for healthcare providers. Such training would not only enhance their diagnostic proficiency but also equip them with the necessary skills to implement effective care protocols for patients at risk of delirium [42]. Another important point that nurses have repeatedly mentioned is the frequency of assessment. According to the requirements of the scale [43], at least once every 8 h, but the such frequency will cause problems. Nurses worry that delirium assessments may embarrass patients, families, and professionals, and some patients show signs of frustration and anger [44–46]. The frequency of cognitive assessments is a topic of ongoing debate among healthcare stakeholders. However, the evidence indicates that alterations in cognitive status or behavior should serve as a primary indicator for assessment. It is more logical to conduct assessment in response to changes in a patient’s condition, employing a dynamic approach to selecting the timing of assessments. This practice diverges from the conventional, prescriptive use of standardized assessment tools, which may not always align with the individual needs of patients experiencing cognitive fluctuations.

In addition, with the development of enhanced recovery after surgery [47], nurses pay more attention to the implementation of early activities for patients [48]. Patients can get out of bed after surgery, which for the state of consciousness and cognition is a great help. Therefore, the nursing staff reconsiders whether it is unnecessary to assess the frequency required according to the assessment table. Not only do nurses need a tool for assessing delirium, but they also need to develop programs or policies for using it, such as using it as part of a medical record, standard care processes, electronic assessments, etc [49, 50].

With the aging process in China, some pension problems are becoming more and more prominent, and many elderly people living alone. These cognitive problems are often neglected by caregivers, who consider some behaviors of the elderly as changes caused by normal aging [50, 51]. These slow changes, coupled with the acute trauma of the disease, can easily lead to delirium [52–54]. Delirium is just one of the most important nursing problems in this study. Besides, more nursing problems suggest that we should pay attention to, such as delirium and dementia, depression, patients after discharge of continuing care, and so on. Nurses gradually can find out from the adverse events that the delirium state of the patient led to the occurrence of nursing incompatibility and extubation, and then analyze the risk factors that might lead to delirium. Furthermore, family members often fail to understand the patient’s acute cognitive function changes, and through the assessment of the situation, we can explain their questions in detail, obtain family support, and also guide family members to increase confidence in the rehabilitation of patients [55–57]. All these are conducive to the management of delirium patients in the later stage.

Limitations and implication for future studies.

The present study acknowledges several intrinsic limitations that require attention. Firstly, the elderly in this study are located in a specialist area of post-surgery or intensive care. the limited sample size, though suitable for qualitative inquiry, may restrict the generalizability of the results. Secondly, the exclusion of geriatricians from the interview process represents a notable limitation, as it omits the critical insights of specialists in the field. Their expertise is particularly valuable for understanding the nuances of conditions in elderly patients, which could significantly augment the applicability and relevance of the study’s findings. Thirdly, the sampling process, while meticulously executed, may be perceived as lacking objectivity due to the pre-existing familiarity with each participant prior to the commencement of interviews. Despite the assignment of numeric codes to ensure anonymity, the selection criteria for interviewees, which included the capacity to perform assessments post-training in delirium assessment, could potentially introduce a bias. This selection bias might skew the representativeness of the sample, affecting the generalizability of the findings and necessitating a cautious interpretation of the results within the confines of the study’s specific context. In anticipation of future research, it is imperative to expand the scope and depth of delirium assessments. This should include incorporating a more diverse and extensive sample size to enhance the robustness of the study. Additionally, the inclusion of geriatricians’ perspectives will be essential to provide a comprehensive understanding of delirium in the elderly. By refining the methodology and enriching the content of these assessments, the efficacy and responsiveness of delirium assessments can be improved, ultimately serving to better address the unique needs of geriatric patients and amplifying the study’s impact within the scientific community.

## Conclusions

In conclusion, geriatric nursing specialists face the intricate and diverse challenges inherent in caring for the elderly, with delirium emerging as a significant concern that warrants focused attention. The ongoing refinement of delirium assessment methodologies is expected to substantially improve the caliber of care delivered to this vulnerable population. Nonetheless, the complexities encountered during the assessment process require thorough examination. The discrepancies observed in current delirium assessments, particularly in the selection and application of content and methodologies, are striking.

### Implications

The findings in this study highlight an urgent requirement for the development of innovative strategies to overcome these obstacles. It is imperative that clinical practice incorporates a dynamic approach to patient evaluation, initiating assessments following an initial risk factor screening. This approach should be sensitive to regional differences and adapted to the unique attributes of the elderly patient cohort. Additionally, it is recommended that future research and clinical initiatives concentrate on enriching both the content and the assessments methodology of delirium. This may ensure that these tools are more efficacious and attuned to the specific needs of geriatric patients, thereby facilitating a more nuanced and responsive care paradigm.

Furthermore, a more insightful approach to understanding the intricacies of delirium care may involve a comprehensive exploration of the lived experiences of nurses and the dynamics of facilitators and barriers encountered by both healthcare professionals and families. To achieve this, employing a combination of innovative research methodologies is proposed. Specifically, Online photovoice (OPV) offers a platform for participants to visually articulate their experiences, providing a rich, contextually grounded dataset [58, 59]. This is complemented by Online Interpretative Phenomenological Analysis (OIPA), which delves into the subjective interpretations and personal meanings attributed to these experiences, thereby offering a profound understanding of the psychological landscape of delirium care [60]. Additionally, incorporating Community-Based Participatory Research (CBPR) ensures that the research is not only academically rigorous but also practically relevant [61]. This approach emphasizes collaboration with the community, ensuring that the research is shaped by the insights and needs of those directly involved in managing delirium. The integration of these methodologies is anticipated to yield a multidimensional perspective that respects the complexity of the clinical environment and the lived realities. This methodological synergy is expected to illuminate the nuances of delirium management, offering a robust framework for developing evidence-based interventions and policies that are both responsive to the needs of patients and caregivers and grounded in the realities of clinical practice.

### Electronic supplementary material

Below is the link to the electronic supplementary material.

Supplementary Material 1

Supplementary Material 2

Supplementary Material 3

## Data Availability

All data generated or analyzed during this study are included in this published article.
