# Supplementary material for: A qualitative study of geriatric specialist nurses’ experiences to navigate delirium in the elderly
Source: BMC Nurs. 2024 Jun 25;23:426. doi: 10.1186/s12912-024-02100-x (PMC11197179; doi:10.1186/s12912-024-02100-x)
Supplement: Supplementary file 1 — Supplementary Material 1 [file 12912_2024_2100_MOESM1_ESM.docx]

**Huadong hospital**

**Delirium assessment record sheet**

**Name： age： gender： bed number： Hospitalization number： diagnosis：**

**Mode of anesthesia: whether general anesthesia yes no type of surgery：**

**Date of surgery： Time to return to ward：**

|  | Preoperative score | score（0-2 points） | | | | | | | | |
| --- | --- | --- | --- | --- | --- | --- | --- | --- | --- | --- |
| Time point  Symptoms |  |  |  |  |  |  |  |  |  |  |
|  |  |  |  |  |  |  |  |  |  |  |
| **I.** **Disorientation**  Showing inability to distinguish between time or place or the identity of others around them. |  |  |  |  |  |  |  |  |  |  |
| **II.** **Abnormal behavior**  For example: pulling a catheter or dressing on the body, or trying to get out of bed and similar behavior. |  |  |  |  |  |  |  |  |  |  |
| **III.** **Abnormal speech communication**  It is characterized by incoherence, silence and making absurd or inexplicable speeches. |  |  |  |  |  |  |  |  |  |  |
| **IV.** **Delusions / hallucinations**  To see or hear something that does not exist; distort the vision. |  |  |  |  |  |  |  |  |  |  |
| **V.** **Psychomotor retardation**  For example, the patient is slow to respond to pain and / or cannot be awakened. |  |  |  |  |  |  |  |  |  |  |
| **Total score** |  |  |  |  |  |  |  |  |  |  |
| **Nurse in charge** |  |  |  |  |  |  |  |  |  |  |

Note:

1. If the preoperative score is less than 2 points, then proceed with postoperative assessment.

2. A total score of 2 points or more indicates a positive delirium.

3. The postoperative delirium assessment record should be written according to the actual surgical situation, with at least 8 hours per session, for a total of 9 sessions.

4. If delirium persists or occurs after more than 72 hours, continue with the assessment.
