# Supplementary material for: A qualitative study of geriatric specialist nurses’ experiences to navigate delirium in the elderly
Source: BMC Nurs. 2024 Jun 25;23:426. doi: 10.1186/s12912-024-02100-x (PMC11197179; doi:10.1186/s12912-024-02100-x)
Supplement: Supplementary file 2 — Supplementary Material 2 [file 12912_2024_2100_MOESM2_ESM.docx]

**Huadong hospital**

**ICU Delirium assessment record sheet**

**Name： age： gender： bed number： Hospitalization number： diagnosis：**

**Mode of anesthesia: whether general anesthesia yes no type of surgery：**

**Date of surgery： Transfer time to ICU：**

**Transfer out ICU time:**

| Item | Evaluation record | |
| --- | --- | --- |
| Feature 1: acute changes or fluctuations in the state of consciousness |  |  |
| Is the patient's state of consciousness different from the baseline?  Or have there been any fluctuations in the RASS score in the past 24 hours? |  |  |
| Feature 2: attention disorder |  |  |
| The numerical method checks the attention and instructs the patient to pinch his hand when reading "8". Read the following numbers aloud in a normal tone, each with an interval of 3 seconds.6 8 5 9 8 3 8 8 4 7 |  |  |
| Feature 3: change in the level of consciousness |  |  |
| If the current score of RASS is not 0, it is positive. |  |  |
| Feature 4: confusion of thinking |  |  |
| Right and wrong:  1. Can a stone float on the water?  2. Are there any fish in the sea?  3. 1 jin heavier than 2 jin?  4. Can you hammer a nail with a hammer?  Execute instructions: say to the patient, "hold out these two fingers" (the examiner demonstrates) and then say, "now hold out the same number of fingers with the other hand" (the examiner does not demonstrate) if the patient cannot successfully execute all instructions, record an error. |  |  |
|  | Nursing in charge |  |
| RASS score | | |
| Score |  |  |
| +4 | Aggression | Aggressive behavior, violence, immediately pose a danger to the staff |
| +3 | Extremely restless | Pull or pull out various pipes or intubations; aggressive |
| +2 | restless | Frequent aimless movements, resistance to the ventilator |
| +1 | inattentive | Anxiety, fear, and movement are not aggressive |
| 0 | Sober and calm | Actively pay attention to the caregiver |
| -1 | Lethargy | Not fully awake, but can remain awake after sound stimulation (open eyes and eye contact > 10 seconds) |
| -2 | Mild sedation | Can maintain a brief awake state after sound stimulation (eye opening and eye contact < 10 seconds) |
| -3 | Moderate sedation | Movement or eye-opening reaction after sound stimulation (but no eye contact) |
| -4 | Deep sedation | Do not respond to sound stimulation, but move or open your eyes after physical stimulation |
| -5 | Don't wake up. | No response to sound or physical stimulation |

Note:

1. If more than 3 characteristics are positive, it is considered a positive delirium.
2. The postoperative delirium assessment record should be written according to the actual surgical situation, with at least 8 hours per session.
