# Supplementary figures and images for: A qualitative study of geriatric specialist nurses’ experiences to navigate delirium in the elderly
Source: BMC Nurs. 2024 Jun 25;23:426. doi: 10.1186/s12912-024-02100-x (PMC11197179; doi:10.1186/s12912-024-02100-x)

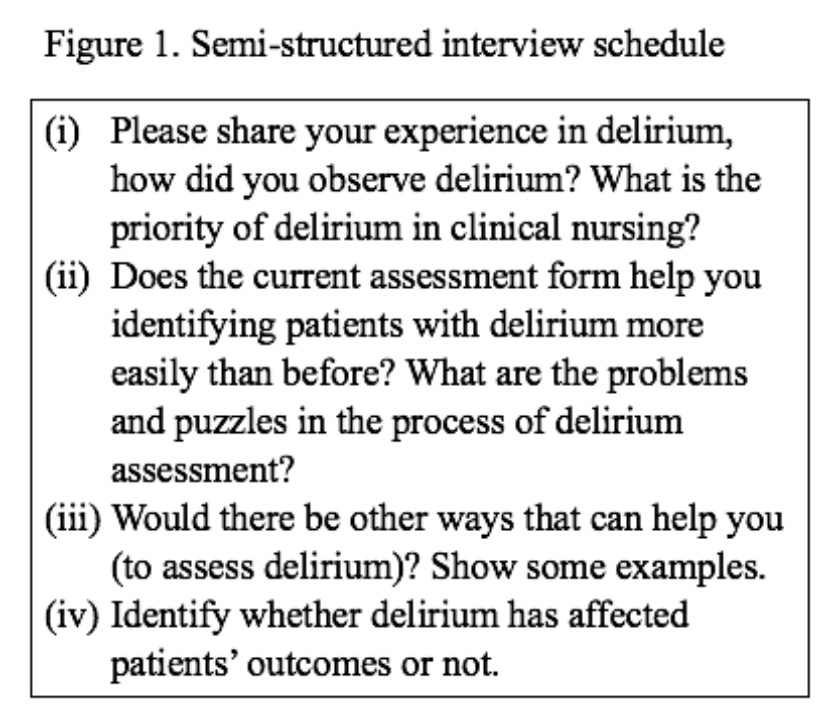

Supplement: Supplementary file 3 — Supplementary Material 3 [file 12912_2024_2100_MOESM3_ESM.jpg]
